# (TG/CA)_n _repeats in human gene families: abundance and selective patterns of distribution according to function and gene length

**DOI:** 10.1186/1471-2164-6-83

**Published:** 2005-06-03

**Authors:** Vineet K Sharma, Samir K Brahmachari, Srinivasan Ramachandran

**Affiliations:** 1 G.N. Ramachandran Knowledge Centre for Genome Informatics, Institute of Genomics and Integrative Biology, Mall Road, Delhi 110 007, India

## Abstract

**Background:**

Creation of human gene families was facilitated significantly by gene duplication and diversification. The (TG/CA)_n _repeats exhibit length variability, display genome-wide distribution, and are abundant in the human genome. Accumulation of evidences for their multiple functional roles including regulation of transcription and stimulation of recombination and splicing elect them as functional elements. Here, we report analysis of the distribution of (TG/CA)_n _repeats in human gene families.

**Results:**

The 1,317 human gene families were classified into six functional classes. Distribution of (TG/CA)_n _repeats were analyzed both from a global perspective and from a stratified perspective based on their biological properties. The number of genes with repeats decreased with increasing repeat length and several genes (53%) had repeats of multiple types in various combinations. Repeats were positively associated with the class of Signaling and communication whereas, they were negatively associated with the classes of Immune and related functions and of Information. The proportion of genes with (TG/CA)_n _repeats in each class was proportional to the corresponding average gene length. The repeat distribution pattern in large gene families generally mirrored the global distribution pattern but differed particularly for *Collagen *gene family, which was rich in repeats. The position and flanking sequences of the repeats of *Collagen *genes showed high conservation in the Chimpanzee genome. However the majority of these repeats displayed length polymorphism.

**Conclusion:**

Positive association of repeats with genes of Signaling and communication points to their role in modulation of transcription. Negative association of repeats in genes of Information relates to the smaller gene length, higher expression and fundamental role in cellular physiology. In genes of Immune and related functions negative association of repeats perhaps relates to the smaller gene length and the directional nature of the recombinogenic processes to generate immune diversity. Thus, multiple factors including gene length, function and directionality of recombinogenic processes steered the observed distribution of (TG/CA)_n _repeats. Furthermore, the distribution of repeat patterns is consistent with the current model that long repeats tend to contract more than expand whereas, the reverse dynamics operates in short repeats.

## Background

The evolution of organisms with increasing complexity was significantly facilitated by duplication of genes and genomes followed by diversification [1,2]. Gene duplication *per se *produces two identical copies. Subsequently, one of the copies may either accumulate beneficial changes to give rise to a functionally diversified gene or accrue deleterious mutations to end up as a pseudogene, while the other copy retains its original function. The former mechanism leads to the creation of 'gene families' capable of carrying out diverse functions [2,3].

The classification of genes into gene families by Human Gene Nomenclature Committee (HGNC) on the basis of sequence similarity of the encoded proteins [4] and the availability of human genome sequence [5] allow us to carry out a comprehensive survey of a class of important functional element, namely the (TG/CA)_n _repeats. Analysis of the distribution of (TG/CA)_n_repeats within genes in 'present day' gene families holds the potential to provide insights into the factors steering their abundance and selective distribution. Although the characteristic property of (TG/CA)_n _repeats exhibiting length polymorphism has been widely used in genetic mapping [6], a growing body of evidence accumulating over several years point to their multiple functional roles in various biological processes.

The (TG/CA)_n _repeats have a propensity to undergo structural transitions [7-10] and have been shown to modulate transcription in several genes including rat *α-lactalbumin *[9], rat *prolactin *[11], *MMP-9 *[12], *IFN-γ *[13], *EGFR *[14], *HSD11B2 *[15], tilipia *prolactin1 *[16] and human housekeeping genes [17]. Furthermore, the (TG)_n _tracts have been observed to act as stimulator in recombination and in mRNA splicing [18-22].

In the current study, the analysis of distribution of (TG/CA)_n _repeats in human gene families affords assessment of the distribution of these repeats by examining for positive association or negative association with respect to gene length and function.

## Results

### Characteristics of human gene families and their functional classification

**Figure 1:**
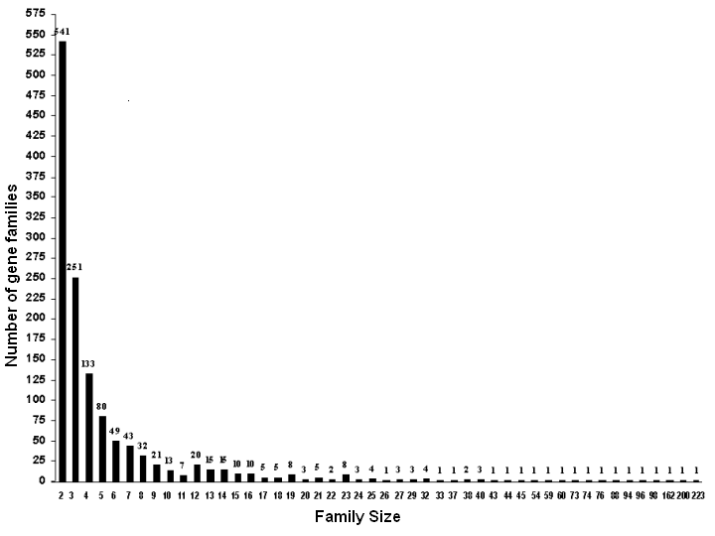
Distribution pattern of human gene families with respect to family sizes. X axis: family size (number of genes in each gene family). Y axis: number of gene families corresponding to various family sizes. Note the inverse exponential relationship.

Each of the 1,317 gene families included members with similar functional roles. The family sizes varied in a wide range between 2 to 223 members (Figure 1). The number of gene families was found to bear an inverse exponential relation to family size. About two-fifths of the gene families were duplex. Only three gene families had more than 100 members per family: Immunoglobulin heavy chain (162 genes), Zinc finger proteins (200 genes) and Solute carrier (223 genes).

**Figure 2:**
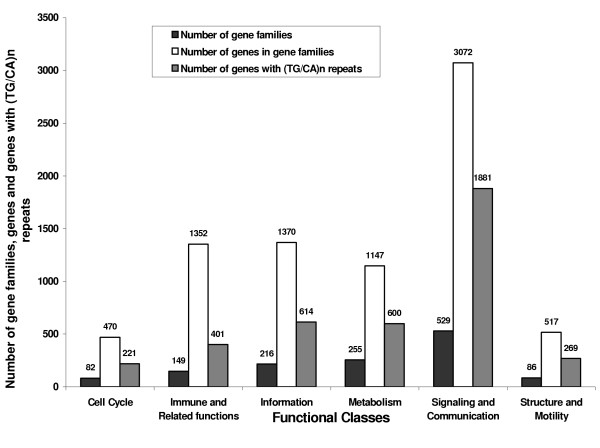
Global distribution of gene families, genes and proportion of genes containing (TG/CA)_n _repeats classified into the six functional classes. The numbers correspond to the height of the vertical bars in each group.

The functional classification of 1,317 gene families comprising 7,928 genes in the six functional classes unveiled that the Signaling and communication is largest with 529 families and 3,072 genes (Figure 2). The Cell cycle is the smallest with 82 families and 470 genes.

Of the 1,317 gene families, 131 were entirely intrachromosomal. Chromosome 1 had the largest number with 17 families followed by chromosomes 19 and 11 with 13 and 12 families respectively. The remaining chromosomes had less than 10 intrachromosomal gene families per chromosome. The functional classification of these 131 intrachromosomal gene families revealed that the highest number (45) belonged to the class of 'Immune and related functions' closely followed by the class of Signaling and communication with 40 families. The remaining classes had the following distribution of gene families: Metabolism (24), Information (15), Structure and motility (5) and Cell cycle (2). These observations indicate that the creation of intrachromosomal human gene families was driven by large number of duplications followed by divergence in selected functional classes.

### Global distribution of (TG/CA)_n _repeats (n ≥ 6 units) in gene families

Of the 1,317 gene families, 732 families had (TG/CA)_n _repeats in at least one of their members and 326 families had repeats in all their members. Of the 7,928 genes in 1,317 families, 3,986 genes had intragenic (TG/CA)_n _repeats of length greater than or equal to 6 units. All 3,986 genes had repeats in their introns. Only 277 genes had (TG/CA)_n _repeats in exons indicating that these repeats are mainly present in introns.

The distribution of genes with (TG/CA)_n _repeats in the six functional classes is displayed in Figure 2. It is apparent that the class of Signaling and communication had the highest number of genes with (TG/CA)_n _repeats. Comparison of the proportion of genes with repeats in each class with the global proportion showed that the class of Signaling and communication had significantly higher than the expected proportion (p < 0.0001, Binomial test). In contrast, the classes of Immune and related functions and Information had significantly lower than the expected proportion of genes with repeats (p < 0.0001 and p < 0.0002 respectively). The proportion of genes with repeats was not significantly different from the global proportion in the Cell cycle, Metabolism and Structure and motility classes. These observations show that the (TG/CA)_n _repeats exhibit positive association with the genes belonging to Signaling and communication whereas, they are negatively associated with the genes belonging to Immune and related functions and Information.

It has been shown that the human genome has an isochore structure that varies in GC content [5]. This variation raises the possibility that the observed selective distribution of (TG/CA)_n _repeats might have arisen due to fluctuations in the local %(G+C) content of the genomic region as opposed to function. We examined this by comparing the average %(G+C) content of the genes in the six functional classes with the corresponding proportions of genes with repeats. The average %(G+C) content was observed to be in the narrow range (47–49%) in the six functional classes whereas, the proportion of genes with repeats varies widely in the range 29.6–61%. These observations indicate that the proportion of genes with repeats is significantly determined by function instead of small fluctuations in %(G+C) content.

### Correlation between gene length, function and global distribution of (TG/CA)_n _repeats

**Figure 3:**
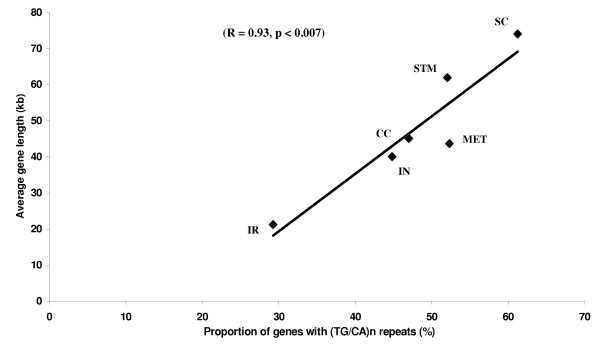
Relationship between proportion of genes with (TG/CA)_n_ repeats in each functional class and the average gene length in the corresponding functional classes.  X axis: Proportion of genes with (TG/CA)_n_ repeats (%); Y axis: Average gene length (kb)  (CC: Cell cycle; IN: Information; IR: Immune and related functions; MET: Metabolism; SC: Signaling and communication; STM: Structure and motility)

Comparison of the proportion of genes containing (TG/CA)_n _repeats with the average lengths of genes in each of the six functional classes revealed a linear relationship (Figure 3, correlation coefficient R = 0.93, p < 0.007). The signaling and communication class had the longest average gene length (74.07 kb) along with the highest proportion of genes with (TG/CA)_n _repeats (61.23%). The class of Immune and related functions had the shortest average gene length (21.26 kb) with the lowest proportion of genes with (TG/CA)_n _repeats (29.65%). These observations show that the proportion of genes with (TG/CA)_n _repeats bears a linear relationship to the length of genes.

### Trinity of (TG/CA)_n _repeats in gene families

**Figure 4:**
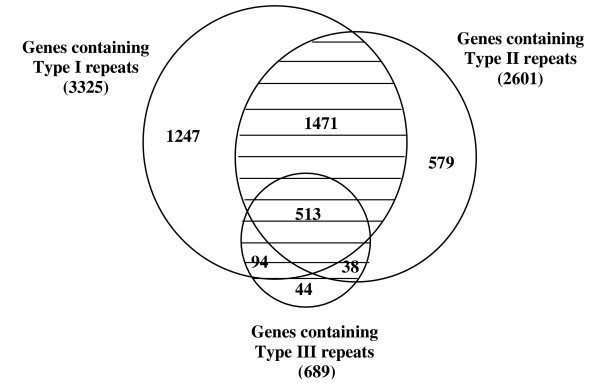
A Venn diagram of the genes with trinity of intragenic (TG/CA)_n _repeats (type I, II and III). Note that several genes (shaded area) have multiple types of repeats in various combinations.

In order to examine the characteristics of distribution of (TG/CA)_n _repeats with respect to multiple functional roles principally governed by their length, we analysed the repeats stratified into three categories: type I (6 ≤ n < 12), type II (12 ≤ n < 23) and type III (n ≥ 23). The results are displayed in Figure 4. The number of genes containing (TG/CA)_n _repeats decreases with increasing repeat length. It is also apparent that several genes (53% of the total) have multiple types of repeats in various combinations.

**Figure 5:**
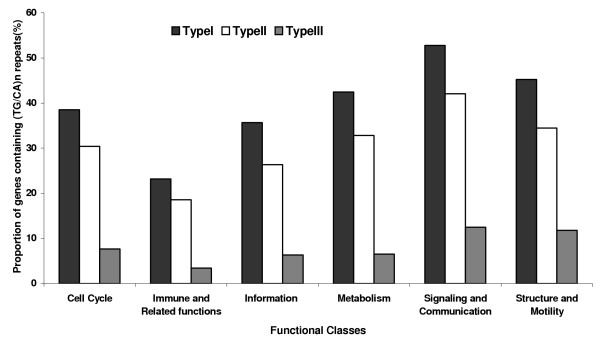
Distribution of proportion of genes with three types of (TG/CA)_n _repeats in the six functional classes.

Classification of the distribution of genes with (TG/CA)_n _repeats stratified into three categories into six functional classes is shown in Figure 5. It is evident that the proportion of genes containing repeats decreases in the order I > II > III in all classes. The proportion of genes containing (TG/CA)_n _repeats of Signaling and communication were significantly higher than the expected proportion in all three categories of repeats (p < 0.0001, type I, II and III). On the other hand, the proportion of genes with (TG/CA)_n _repeats of Immune and related functions and Information were significantly lower than expected proportion in all three categories: Immune and related functions (p < 0.0001, type I, II and III), Information (p < 0.0001, type I and II, p < 0.004, type III). The proportion of genes with type III repeats was marginally lower than the expected proportion in Metabolism class (p < 0.01) and marginally higher than the expected proportion in Structure and motility class (p < 0.02). The proportion of genes with repeats in the three categories was not significantly different from the expected value in the class of Cell cycle. These observations show that repeats of all types are positively associated with the genes of Signaling and communication whereas they are negatively associated with the genes of Immune and related functions and Information.

**Figure 6:**
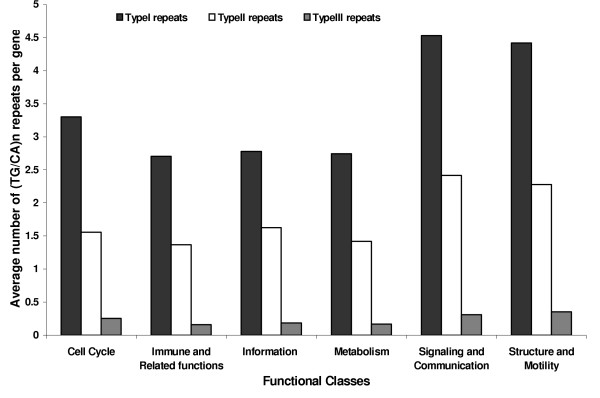
Distribution of the densities of three types of (TG/CA)_n _repeats in the genes of six functional classes.

The distribution of average number of (TG/CA)_n _repeats per gene in the three categories in the six functional classes is displayed in Figure 6. Comparison of the average number of repeats per gene in the three categories with the global distribution pattern revealed that in most cases the observed number was significantly lower than the expected value, except for the genes belonging to Signaling and communication and Structure and motility, which had significantly higher average number of repeats per gene than the expected value (p < 0.0004 in all three categories, both classes). The average number of type III repeats per gene in the class of Cell cycle was not significantly different from the expected value. These observations show that the repeat densities were higher in the genes belonging to Signaling and communication and Structure and motility classes whereas, the genes belonging to other classes had lower repeat densities.

### Large gene families

As a special case of this study, we examined the distribution of (TG/CA)_n _repeats in the top 2% large families (27). The family sizes of this category varied widely from 32 to 223 members. Functional classification of these large families revealed the following distribution: Immune and related functions (9), Signaling and communication (8), Information (6), Metabolism (2), Structure and motility (1) and Cell cycle (1).

The proportion of genes with (TG/CA)_n _repeats in large families is displayed in Table 1. Comparison with the global distribution showed that the proportion of genes with repeats was significantly higher than expected value in the Signaling and communication and Structure and motility classes (p < 0.0001, Binomial test). There was no significant difference between the observed and the expected proportion of genes with repeats in the class of Metabolism. In the remaining classes, the proportion of genes with repeats was significantly lower than the expected value (p < 0.0001, Binomial test). As observed with all gene families, a linear relationship was observed between gene lengths and proportion of genes with (TG/CA)_n _repeats (correlation coefficient R = 0.79, p < 0.0001).

**Table 1:** Distribution of (TG/CA)_n _repeats in large gene families

**Functional class and gene families**	**Chromosomal Distribution**^a^	**Average Gene Length (kb)**^b^	**Genes in the family**	**Proportion of Genes with (TG/CA)_n _repeats**	**Number of Genes with (TG/CA)_n _repeats in three categories**			**Average number of repeats per gene in three categories**		
					
					**Type I **	**Type II**	**Type III**	**Type I**	**Type II**	**Type III**
**Cell cycle class**^c^**(1)**										
Histone proteins family	Dispersed	3.55	76	7.9	4	4	0	1.7	0.7	0
**Immune and related functions class (9)**										
Interleukins	Dispersed	14.67	43	32.6	9	6	2	1.3	0.9	0.1
Serine (or cysteine) proteinase inhibitor family	Dispersed	17.87	32	50	12	9	2	1.4	0.8	0.2
Tumor necrosis factor (ligand) superfamily	Dispersed	23.49	38	63.2	21	14	1	1.9	0.9	0.1
CD antigens	Dispersed	26.96	54	46.3	20	11	3	1.9	1.3	0.3
Immunoglobulin heavy chains	Intrachromosomal	0.38	162	0.6	1	0	0	1	0	0
Immunoglobulin kappa chains	Intrachromosomal	0.55	73	5.5	1	3	0	0.3	3	0
Immunoglobulin lambda chains	Intrachromosomal	0.35	88	0	0	0	0	0	0	0
Interleukin receptors family	Dispersed	29.77	32	59.4	17	10	0	3.4	0.9	0
T cell receptor beta chains	84 Intrachromosomal, 9 Dispersed	0.42	94	9.6	5	3	1	0.8	0.3	0.1
**Information class (6)**										
Homeo box	Dispersed	5.48	40	25	6	6	1	1.2	0.9	0.2
Eukaryotic translation initiation factor	Dispersed	36.54	33	45.5	13	10	3	2.1	0.9	0.2
Zinc finger protein family	Dispersed	30.33	200	42.5	63	44	6	2.2	1.1	0.1
DEAD/H (Asp-Glu-Ala-Asp/His) box polypeptides	Dispersed	43.44	32	62.5	17	8	2	1.5	0.6	0.1
Ribosomal protein genes	Dispersed	4.94	96	6.3	6	0	0	1	0	0
Mitochondrial ribosomal protein genes	Dispersed	16.92	74	23	14	12	1	1.8	0.9	0.1
**Metabolism class (2)**										
Cytochrome P450 superfamily	Dispersed	31.34	45	46.7	15	11	2	1.9	1	0.1
Proteasome subunit genes	Dispersed	25.64	40	32.5	11	8	0	1.5	0.8	0
**Signaling and Communication class (8)**										
G protein-coupled receptor family	Dispersed	24.71	98	33.7	26	19	6	2.3	1.5	0.2
Tripartite motif-containing family	Dispersed	29.29	40	60	19	12	2	1.6	0.8	0.1
Solute carrier family	Dispersed	59.19	223	62.8	134	87	22	2.9	1.5	0.2
RAS oncogene family	Dispersed	39.92	60	65	38	17	4	1.8	0.7	0.1
ATP-binding cassette transporters gene family	Dispersed	73.85	44	68.2	29	24	4	3.6	2.5	0.3
Guanine nucleotide binding protein (G protein) polypeptide genes	Dispersed	58.83	32	59.4	18	12	5	3.3	1.9	0.4
Potassium voltage-gated channel genes	Dispersed	104.95	38	57.9	17	16	6	8.6	4.4	0.5
Protein phosphatase subunit genes	Dispersed	65.62	59	57.6	27	22	7	2.9	1.8	0.3
**Structure and motility class (1)**										
Collagen family	Dispersed	132.83	37	86.5	29	23	10	5.7	2.3	0.4

^a^: Chromosomal distribution of the members of gene families. 'Dispersed' indicates that members are distributed on different chromosomes.

^b^: average gene length (in kb) for each gene family.

^c^:Numbers in parentheses show the number of large sized gene families in each functional class.

The large *Collagen *gene family belonging to the class of Structure and motility had the highest proportion of genes containing repeats (86.5%). In order to analyze this further, we examined the sequence conservation of the region flanking 200 bases upstream and downstream in addition to the repeats by comparing the human sequence with the available genome sequence of Chimpanzee (*Pan troglodytes*), a nearest ancestor to human [46]. We observed, that of the 268 sequence segments including repeats from human *Collagen *genes, 244 were conserved with greater than 92% identity in the chimpanzee. Of these 244 repeats in human *Collagen *genes, 73 repeats were identical in length, 142 repeats displayed length polymorphism in the chimpanzee, 27 repeats had point mutations and in 2 cases there were no repeats in the corresponding segments in the chimpanzee. These observations show that both human and chimpanzee *Collagen *genes have high repeat content, high conservation of position and flanking sequences of the repeats. However, majority of repeats exhibited length polymorphisms, which is consistent with their characteristic property [6].

## Discussion

The inverse relationship between the number of gene families and their corresponding sizes, resulting in a large number of small sized gene families, suggests that several duplicated copies may have been lost during the first round of genome duplication itself, considering the hypothesis of two rounds of genome duplication in vertebrate evolution [1,23,24]. The non-uniform distribution of the number of gene families across the six functional classes suggests that widespread gene duplication across gene families spanning a wide range of functions may have been less productive in attaining higher levels of complexity. An alternate course involving large amount of duplications followed by divergence producing a wide range of functions in selected classes might have been favorable. The support for the latter hypothesis emerges from the fact that large sized gene families, inherently low in number, mainly belong to Immune and related functions (required to tackle a wide range of infections), Signaling and communication (required to respond to diverse environmental stimuli) and Information class (required to implement complex molecular processes through supramolecular assemblies or organelles). A few members of large sized gene families of Metabolism class function in bioenergetics and xenobiotic metabolism and of Cell cycle class function in packaging of nuclear DNA. Similarly the large *Collagen *gene family of Structure and motility class offers a useful repertoire for the formation of multiple tissues [25].

It is apparent that short repeats are abundant in human genes and long repeats are rare. Our findings are consistent with the observations by Whittaker et al. (2003), who showed that longer repeats are more likely to contract than expand [47]. Accordingly, contraction of long repeats in time would result in accumulation of higher number of short repeats.

Of the six functional classes, the Signaling and communication class was the richest in repeats including the proportion of genes with repeats and repeat densities. Many of the genes belonging to this class function at the interface between the body and its environment that appears to be a distinct feature of eukaryotes [28] to confer species-specific advantages [24,41]. The positive association of (TG/CA)_n _repeats associated with genes of this class strongly argues for a positive temporal regulatory role that could provide for variations in gene expression to complement the enormous diversity characteristic of this class. Compared to this class, the anciently evolved gene families of Information and Cell cycle are poor in repeats. Considering the fact that these genes are highly conserved [28-30] and are involved in implementing the molecular processes acting at the core of cellular physiology, these observations suggest that repeats are negatively associated with these genes to avoid unpredictable consequences for the normal functioning of the cell.

Another argument in favor of these inferences stems from the linear relationship between the average gene length of gene families belonging to the respective functional classes and the proportion of genes with repeats in these classes. The average length of genes belonging to Information class was short and this factor aids in obtaining high levels of expression of these genes [29]. This requirement, however, generates a space constraint to accommodate additional elements. This situation contrasts to that of genes of Signaling and communication class with higher average gene length offering more space for accommodating other regulatory elements. The analysis of *Collagen *gene family belonging to large sized families presents itself as an interesting case. Most of the members of this family have (TG/CA)_n _repeats. Sequence comparisons of repeat containing regions of human *Collagen *genes with the nearest ancestor to humans, the Chimpanzee, revealed that although there is high conservation in terms of content and position of repeats, majority of repeats were polymorphic, which is consistent with their characteristic property [6]. Among repeats that displayed polymorphism between human and Chimpanzee, nearly equal proportions of human repeats were either contracted or expanded in Chimpanzee. These results are also consistent with the Whittaker's model [47].

Strikingly, the genes of Immune and related functions class are poor in (TG/CA)_n _repeats in general and in type III repeats in particular. A characteristic trend of this class is to have large sized families with their genes arranged juxtaposed on the same chromosomal locations. This arrangement increases the possibility of these gene families to display more uniform sequence characteristics [31]. Further, these genes have the smallest average gene length indicating a compact arrangement, which is likely to act as a space constraint in the accommodation of (TG/CA)_n _repeats. In addition, the negative association of type III (TG/CA)_n _repeats in these genes may have a directional role. The immunoglobulin genes use the 7 bp and 9 bp repeats for generation of variants through VDJ recombination [33]. Accommodation of type III (TG/CA)_n _repeats (n ≥ 23) might introduce variations in this process and could result in loss of directional recombination essential to generate diversity in immunoglobulins and T cell receptor chains in an ordered manner.

## Conclusion

The (TG/CA)_n _repeat distribution pattern observed in human gene families is consistent with Whittaker's model of repeat expansion and contraction. It appears that multiple factors including gene length, function and directionality of recombination processes steered the observed selective patterns of distribution of (TG/CA)_n _repeats in human gene families.

## Methods

### Sequence retrieval and mapping of (TG/CA)_n _repeats

Sequences of 35,114 human genes (build number 33) were retrieved from LocusLink [43] using a JavaScript program. A sum of 192 genes could not be retrieved because of either inaccessibility to the LocusLink page or absence of the link for retrieving the gene sequence. A gene in this analysis is considered as the nucleotide sequence from the start of first exon to the end of last exon. If alternate splicing was reported, the gene length considered was the start of first exon to the last known exon including all alternatively spliced products for that gene.

Perl scripts, '*SimRep*' and '*RepGene*' were written for the identification and mapping of perfect intragenic (TG/CA)_n _repeats of length n ≥ 6 units in genes [17]. Throughout this work we have used n ≥ 6 units as the minimum cut-off to identify (TG/CA)_n _repeats. All repeats were scored in the intragenic region (exons and introns only).

### Categorization of (TG/CA)_n _repeats

We grouped (TG/CA)_n _repeats into three categories (types I, II and III), according to their length and biological properties. Type I (TG/CA)_n _repeats, in the range 6 ≤ n <12 units, are short repeats based on the observation that a repeat length of 8 units (n = 8) is minimum to be likely polymorphic [34,35]. Type II (TG/CA)_n _repeats comprise of 12 ≤ n < 23 units and is based on the observation that more than 93% of the (CA)_n _repeats of n ≥ 12 units are polymorphic [6]. Further, repeats of this length have also been shown to have preferential binding to nuclear factors compared to short repeats [36] and can also stimulate mRNA splicing [21,22]. Type III repeats consist of relatively long reiterations of (TG/CA)_n _(n ≥ 23 units) and have propensity to adopt structures such as Z DNA [8,9,37]. Other studies have shown that (TG/CA)_n _repeats of length greater than 22.5 units can stimulate recombination [18-20].

### Clustering of genes into gene families

Functional roles of a large number of human genes are not well known. Presently, these genes are assigned hypothetical annotations. Genes labeled as 'LOC', 'DFKZP', 'FLJ', 'HSPC', 'HSU', 'HT', 'KIAA', 'ORF', 'hypothetical', 'PRO' and 'pseudogenes' without clear functional details were filtered out. A total of 22,688 genes were removed in this filtering exercise. Out of the remaining 12,426 genes, a total of 8,778 genes (25% of total) were clustered into gene families based on their gene root symbols as defined in the guidelines of Human Gene Nomenclature Committee (2002) [4]. The remaining 3,648 genes could not be clustered into gene families and are solitary.

The HGNC guidelines consider sequence and functional similarity of proteins encoded by genes while grouping them into gene families [4,38,39]. A root symbol signifies a gene family. The family members are designated by Arabic numerals placed immediately after the gene root symbol, for example *GPR1*, *GPR2*, *GPR3 *for genes of the G protein-coupled receptor family. A Perl script namely *Clustergene *was written to cluster 8,778 human genes into 1,556 gene families. The Perl script called *ChromoCluster *was written to report gene families located on the same chromosome. Subsequently these gene families were classified into the six functional classes as described below.

### Functional Classification of gene families for comparative analysis

The gene families were classified into six functional classes namely, 'Information', 'Cell cycle', 'Metabolism', 'Signaling and communication', 'Immune and related functions' and 'Structure and motility' based on the scheme defined by Adams et al. [40]. We combined the functional classes of replication, transcription, RNA processing and translation into 'Information' class based on Andrade et al. [41].

'Cell cycle' includes cell cycle, apoptosis, chromosomal structure and DNA repair; 'Immune and related functions' includes immunology, homeostasis, carrier proteins/membrane transport and stress response; 'Information' includes protein synthesis, translation factors, ribosomal proteins, post-translational modification/targeting, protein degradation, tRNA synthesis/metabolism, RNA synthesis, transcription factors, RNA polymerase, RNA processing, RNA degradation, DNA synthesis/replication and DNA repair; 'Metabolism' includes amino acids, nucleotides, sugars, lipids, cofactors, protein modification, energy and carrier proteins/membrane transport; 'Signaling and communication' includes receptors, hormone/growth factors, intracellular transducers, effectors/modulators, metabolism, cell adhesion and channels/transport proteins; 'Structure and motility' includes cytoskeletal, microtubule-associated proteins/motors and extracellular matrix.

Assignment of gene families to each of the functional classes was carried out according to their annotations in Gene Ontology [42] and LocusLink [43] databases. Out of the total 1,556 gene families, 1,317 could be classified into any of the six functional classes. The remaining 239 families could not be classified unambiguously due to limited information on gene function. Subsequent analysis, with respect to functional classification and distribution of (TG/CA)_n _repeats, presented here is from 1,317 gene families comprising of 7,928 genes.

### Alignment of human (TG/CA)_n _repeats and flanking sequences with Chimpanzee genome sequence

The repeats present in human *Collagen *genes were aligned with Chimpanzee (*Pan troglodytes*) genome by using 'BLAT' software available at UCSC Genome Bioinformatics Site [49]. Nucleotide segments including the repeats and containing 200 nucleotides upstream of the start and 200 nucleotides downstream from the end of each of the (TG/CA)_n _repeat were extracted for human *Collagen *genes [48]. These segments were aligned with the Chimpanzee genome (Build 1, version 1, Nov 2003) using BLAT. Only those segments that showed more than 92% identity were noted as conserved.

### Statistical methods

Significance of the differences between the proportions of genes containing repeats and repeats densities in the six functional classes compared with global distribution was tested using Binomial proportions test. The observed proportion in each class was tested against the expected proportion, which was computed assuming no preference with respect to function. Correlation coefficient (R) was computed to examine the relationship between average gene length of gene families belonging to a functional class and the proportion of genes with (TG/CA)_n _repeats in the corresponding functional classes. The 'Interactive Statistical Calculation Pages' website  was used to perform the statistical tests.

## Authors' contributions

VKS conceived of the idea, developed algorithms in Perl, carried out the analysis and wrote the manuscript. SKB gave scientific suggestions for improving the quality of the work and participated in manuscript preparation. SR is the group leader, gave scientific suggestions, helped in the statistical analysis, critical examination, presentation, writing and manuscript preparation.
